# Obstruction of the Bowels, and Extra-Uterine Pregnancy

**Published:** 1859-03

**Authors:** R. F. Henry

**Affiliations:** Princeville, Ill.


ARTICLE VIII.

### OBSTRUCTION OF THE BOWELS AND EXTRA-UTERINE PREGNANCY.

BY DR. R. F. HENRY, OF PRINCEVILLE, ILL.

On the 22d ultimo, I was called to see Mrs. T——, who was attacked, a few hours previous to my arrival, with severe pains in the epigastric region, which I diagnosed and treated as colic. Her bowels had acted freely a short time before the attack commenced. There was no flatulent distention of the abdomen, and but little tenderness on using pressure. The use of anodynes and alteratives gave her relief, and in the course of twenty-four hours I administered a cathartic, which I found necessary to repeat in due time, but no action on the bowels followed. A more active purgation was then given, and followed up with clysters, but withal the bowels remained unacted upon. Nausea and vomiting ensued. Pain in the epigastric region returned, with flatulent distention and tenderness. On the third day of the attack, the matters vomited assumed a fæcal character. Dr. L. M. Andrews was then called in, and afterwards Dr. Milligan, of Wyoming, who suggested the possibility of there being intus-susceptio of a portion of the intestines, which might be reached with enema of tepid water. The experiment was tried, but no relief was obtained. To give a full detail of the case seems unnecessary. Suffice it to say, that everything was done to relieve pain, etc., that careful nursing and assiduous attention could effect, but the patient sank and died on the evening of the 27th ult., at 6 o'clock. Eighteen hours after death, the body was examined by Dr. Andrews and myself, Mr. E. Russell being present. On laying open the abdomen, the intestines were seen very much distended and their walls congested. On unraveling the canal, the obstruction was found in the transverse and descending colon; which consisted in a permanent constriction, commencing about midway the transverse and continuing nearly the whole length of the descending colon, the coats of which were at some places atrophied, and at others very much thickened. The whole constricted portion of the colon was empty. On inflating it, we saw it was very small in diameter, varying from half an inch to that of half the natural size. Above the obstruction the intestines were distended by a large quantity of fæcal matter, having no way of escape except by the mouth. A further examination of the contents of the abdomen revealed a peculiar sac, which was situated immediately over the fundus of the uterus, and adherent to a portion of the ilium, omentum and peritoneum, containing a fœtus of three months' growth, and a placenta, both in a pretty good state of preservation. The walls of the sac were, mostly, very thin, composed, apparently, of a mucous, or internal coat, a vascular and serous coats. The base of the sac, however, was thicker, and appeared to be cartilagenous at one point. On examining the connections of the sac more closely, we found another sac or cyst, lying between it and the uterus, and folded upon the latter. Its diameter was about half an inch, its length an inch and a quarter; it was connected at one end with the uterus, the other opened into the sac first described, at its base. The walls of this sac were composed of material similar to that of the uterus itself. The cavity was of sufficient capacity to contain half a teaspoonful of fluids, perhaps, without distention, being gourd-shaped. The small end passed into the cavity of the uterus, at the right superıor angle, near where the fallopian tube arose. The large end communicated with the cavity of the sac, containing the fœtus, by an opening at the end, which very much resembled the os uteri externum.

The uterus, with the exception of this cornu (if I may so call it), was natural in appearance and healthy, having performed its usual function of menstruation, even during the attack of the last sickness.

The ovaries and fallopian tubes, both healthy.

I learn from the surviving husband of deceased, that she was thirty-eight years of age, and had been married nineteen years. Had never given birth to any children, menstruated regularly, and had enjoyed pretty good health until about four years since, when she had an attack of sickness, and at which time I was called to attend her. I did not note the particulars of her case at that time, but I recollect that she had labor pains, or something like them, and that she informed me she was *enciente*, or supposed she was, as she had not had her catamenias for three periods; however, the periodical pains ceased in due time, but were followed by symptoms of peritonitis, which were very obstinate; but she finally got well, and had enjoyed a good share of health until her last illness, except two or three light attacks of colic, which readily yielded to proper remedial agents.

On reviewing this case, it seems to me that the fecundated ovum must have found its way into this *cornu uteri*, when it was developed and expelled, forming a sac in the peritoneum, producing inflammation and adhesion in the contiguous parts, and that this was the cause of her sickness at the time above named, viz: four years ago.

